# Physicians' Perspectives on ChatGPT in Ophthalmology: Insights on Artificial Intelligence (AI) Integration in Clinical Practice

**DOI:** 10.7759/cureus.78069

**Published:** 2025-01-27

**Authors:** Anwar Ahmed, Dalal Fatani, Jose M Vargas, Mohammed Almutlak, Halah Bin Helayel, Rafah Fairaq, Halla Alabdulhadi

**Affiliations:** 1 Research, King Khaled Eye Specialist Hospital, Riyadh, SAU; 2 Oculoplastic and Orbit, King Khaled Eye Specialist Hospital, Riyadh, SAU; 3 Ophthalmology, King Abdullah Bin Abdulaziz University Hospital, Riyadh, SAU; 4 Anterior Segment Division, King Khaled Eye Specialist Hospital, Riyadh, SAU

**Keywords:** artificial intelligence, chatgpt, eye care, ophthalmology, technology in healthcare

## Abstract

To obtain detailed data on the acceptance of an artificial intelligence chatbot (ChatGPT; OpenAI, San Francisco, CA, USA) in ophthalmology among physicians, a survey explored physician responses regarding using ChatGPT in ophthalmology. The survey included questions about the applications of ChatGPT in ophthalmology, future concerns such as job replacement or automation, research, medical education, patient education, ethical concerns, and implementation in practice. One hundred ninety-nine ophthalmic surgeons participated in this study. Approximately two-thirds of the participants had 15 years or more experience in ophthalmology. One hundred sixteen reported that they had used ChatGPT. We found no difference in age, gender, or level of experience between those who used or did not use ChatGPT. ChatGPT users tend to consider ChatGPT and artificial intelligence (AI) as useful in ophthalmology (*P*=0.001). Both users and non-users think that AI is useful for identifying early signs of eye disease, providing decision support in treatment planning, monitoring patient progress, answering patient questions, and scheduling appointments. Both users and non-users believe there are some issues related to the use of AI in health care, such as liability issues, privacy concerns, accuracy of diagnosis, trust of the chatbot, ethical issues, and information bias. The use of ChatGPT and other forms of AI is increasingly becoming accepted among ophthalmologists. AI is seen as a helpful tool for improving patient education, decision support, and medical services, but there are also concerns regarding privacy and job displacement, which warrant human oversight.

## Introduction

Artificial intelligence (AI) has become an increasingly popular tool in the healthcare industry, providing valuable decision support for healthcare providers and assisting with tasks such as diagnosis, treatment planning, and patient monitoring. The use of AI in healthcare has the potential to improve patient outcomes, streamline medical research, and reduce healthcare-related malpractice. However, it is important to carefully consider the limitations and potential biases of AI algorithms and to use them in conjunction with human expertise and clinical judgment [1].

In the field of ophthalmology, AI algorithms are being used to diagnose and treat a variety of eye diseases, including diabetic retinopathy, glaucoma, and age-related macular degeneration. AI algorithms can analyze retinal images and identify signs of these diseases, providing valuable decision support for ophthalmologists and improving the accuracy and efficiency of diagnosis and treatment. While the accuracy of AI algorithms in diagnosing eye diseases is promising, further research is needed to fully understand their potential and limitations in clinical practice [2].

One of the challenges of using AI in healthcare is ensuring use in a safe and responsible manner. The use of AI algorithms in clinical practice raises several legal and ethical concerns, including liability, privacy, and informed consent. Clear regulatory guidelines and transparent algorithm development can help ensure that AI algorithms are used in a safe and responsible manner. Patients should be fully informed on the use of AI algorithms in their care and should have the option to to opt out [1].

Despite the increasing use of AI in healthcare, there is still limited research on the acceptance of AI chatbots among physicians. Chatbots are AI-powered conversational agents that can interact with humans through text or voice. In the context of healthcare, chatbots can be used to provide decision support, answer patient questions, and assist with tasks such as appointment scheduling. Chatbots have the potential to improve patient outcomes and reduce healthcare costs by providing a more efficient and accessible means of healthcare delivery [3]. ChatGPT (OpenAI, San Francisco, CA, USA) for instance, with its huge data can integrate important yet difficult-to-find information for ophthalmologists that could help in patient management, especially in complex cases. Besides, by using AI, chatbots can analyze data and present fruitful findings that save ophthalmologists their time and effort.

Several studies reported the application of ChatGPT in generating medical articles, such as literature reviews, case studies, and research summaries [4]. Researchers have also employed ChatGPT for data extraction, interpretation, and visualization in systematic reviews and meta-analyses. Additionally, ChatGPT has been successfully utilized as a tool for medical education, automated scoring, and other implications in medical academia [5-7].

Physicians have expressed mixed opinions regarding the integration of ChatGPT into medical practice. Some studies reported positive attitudes, with physicians appreciating the tool for its ability to improve the efficiency of certain tasks, such as patient documentation, communication, and information retrieval [8]. Some claim that ChatGPT can even be cited as a coauthor [9]. However, other studies highlighted concerns about data privacy, potential biases in the generated content, and the need for appropriate human oversight [10].

In this study, we aim to evaluate ophthalmologists' perceptions of ChatGPT's impact on clinical decision-making, patient education, and research, with a focus on its utility and ethical challenges.

## Materials and methods

A cross-sectional survey was conducted through an online self-administered-items non-AI generated questionnaire (Appendix 1) between July 13 to August 25, 2023. The study was approved by the institutional review board at King Khaled Eye Specialist Hospital (KKESH) (23069-P). This study adhered to the tenets of the Declaration of Helsinki.

The study participants included ophthalmology consultants, fellows, and residents. We used a convenient sampling method such as snowball to secure a large number of participants. The survey was distributed using professional social media platforms (LinkedIn; Microsoft Corp., Redmond, WA, USA) or direct invitation through text messages or WhatsApp (Meta Platforms, Inc., Menlo Park, CA, USA).

Participation was voluntary, complete anonymity was ensured, and participants provided informed consent. No identifying data were collected and the responses would only be used for the purposes of this study. All completed responses were included.

As the subject is relatively new, there is no available standardized questionnaire testing the acceptance of AI in ophthalmology. Hence, the questionnaire was developed by the authors.

To ensure clarity, a pilot study was conducted and answered by 10 participants. Based on the responses, further modifications to the questionnaire have been made.

The survey included 18 questions about demographic characteristics (age, gender, and nationality), years of ophthalmology practice, and views and opinions of ChatGPT and AI in clinical ophthalmic practice. The survey also included questions about applications of ChatGPT in ophthalmology, future concerns such as job replacement or automation, research, medical education, patient education, ethical concerns, and implementation in practice. AI in medicine was defined as using AI to determine diagnosis and treatment without requiring a doctor.

All completed responses were included and analyzed.

Statistical analysis

Statistical analysis was performed using STATA version 16.0 (StataCorp LLC., College Station, TX, USA). Categorical variables were summarized as frequencies and percentages. Pearson's Chi-squared test was used to evaluate the association between categorical variables. P < 0.05 was considered statistically significant.

## Results

One hundred ninety-nine ophthalmic surgeons participated in this study. Table 1 presents the study demographics. Approximately two-thirds of the participants had 15 or more years of experience in ophthalmology. One hundred sixteen reported that they had used ChatGPT.

**Table 1:** Demographic data and prevalence of ChatGPT use in Ophthalmology (n=199) AI: artificial intelligence; N: number

Age	N (%)
Under 30	31 (15.6%)
30-39	41 (20.6%)
40-49	31 (15.6%)
50-59	47 (23.6%)
Over 60	49 (24.6%)
Gender	
Male	131 (65.8%)
Female	68 (34.2%)
Nationality	
Saudi	70 (35.2%)
Non-Saudi	129 (64.8%)
Experience (in years)	
Less than 5 years	33 (16.6%)
5-10 years	31 (15.6%)
10-15 years	15 (7.5%)
More than 15 years	120 (60.3%)
Ever used (ChatGPT) AI	
Yes	117 (58.8%)
No	82 (41.2%)

According to their use, participants were then divided into two groups: ChatGPT users and non-users to compare the differences in demographics, experience level, views and opinions of ChatGPT use and AI. The comparisons between the two groups are summarized in Table 2. The percentage of males (61%) using ChatGPT was higher than the percentage of females (54%). Participants between the ages of 40 and 49 had the highest percentage (71%) of using ChatGPT. ChatGPT users tend to have a greater belief in the usefulness of ChatGPT and AI in ophthalmology (P=0.001).

**Table 2:** The comparisons between ChatGPT users and non-users regarding demographics, experience level, views, and opinions of using ChatGPT and AI. AI: artificial intelligence; N: number; * Chi-square test; P < 0.05 was considered statistically significant.

	ChatGPT use		Pearson chi2	p-value *
Variable	ChatGPT users N(%)	Non ChatGPT users N(%)		
Age			2.86	0.581
Under 30	19(61.3%)	12 (38.7%)		
30-39	24 (58.5%)	17 (41.5%)		
40-49	22 (71.0%)	9(29.0%)		
50-59	25 (53.2%)	22 (46.8%)		
Over 60	27 (55.1%)	22 (44.9%)		
Gender			0.82	0.366
Male	80 (61.1%)	51 (38.9%)		
Female	37 (54.4%)	31 (45.6%)		
Nationality			0.42	0.516
Saudi	39 (55.7%)	31 (44.3%)		
Non-Saudi	78 (60.5%)	51 (39.5%)		
Experience (in years)			0.73	0.866
Less than 5 years	20 (60.6%)	13 (39.4%)		
5-10 years	19 (61.3%)	12 (38.7%)		
10-15 years	10 (66.7%)	5 (33.3%)		
More than 15 years	68 (56.7%)	52 (43.3%)		
Usefulness			14.33	0.001
Yes	101 (61.6%)	63 (38.4%)		
No	9 (90.0%)	1 (10.0%)		
I do not know	7 (28.0%)	18 (72.0%)		
Efficiency			9.03	0.11
Yes	96 (62.7%)	57 (37.3%)		
No	7 (77.8%)	2 (22.2%)		
I do not know	14 (37.8%)	23 (62.2%)		
The use of AI in Ophthalmology leads to better medical services.			3.48	0.176
Yes	83 (62.9%)	49 (37.1%)		
No	8 (61.5%)	5 (38.5%)		
I do not know	26 (48.2%)	28 (51.8%)		
Ophthalmologists can quickly master the use of AI in medicine.			9.13	0.010
Yes	94 (64.8%)	51 (35.2%)		
No	9 (52.9%)	8 (47.1%)		
I do not know	14 (37.8%)	23 (62.2%)		
Ophthalmologists can easily operate AI in medical settings.			8.18	0.017
Yes	92 (64.8%)	50 (35.2%)		
No	9 (52.9%)	8 (47.1%)		
I do not know	16 (40.0%)	24 (60.0%)		
Expectations of others regarding the potential of AI in ophthalmology			2.39	0.303
Yes	74 (62.7%)	44 (37.3%)		
No	18 (48.6%)	19 (51.4%)		
I do not know	25 (56.8%)	19 (43.2%)		
Expectations among patients Do you think patients are optimistic about the potential of AI in ophthalmology?			1.30	0.522
Yes	52 (63.4%)	30 (36.6%)		
No	21 (53.8%)	18 (46.2%)		
I do not know	44 (56.4%)	34 (43.6%)		
Brand impact Are your views on AI in ophthalmology shaped by the businesses (or brands of such businesses) that developed the AI for ophthalmology?			0.38	0.826
Yes	45 (57.0%)	34 (43.0%)		
No	39 (58.2%)	28 (41.8%)		
I do not know	33 (62.3%)	20 (37.7%)		
knowledge of AI in other contexts			6.71	0.035
Yes	79 (65.8%)	41 (34.2%)		
No	27 (45.8%)	32 (54.2%)		
I do not know	11 (55.0%)	9 (45.0%)		
Knowledge of AI in medicine Do you know much about the use of AI in ophthalmology?			15.94	<0.001
Yes	61 (75.3%)	20 (24.7%)		
No	41 (45.6%)	49 (54.4%)		
I do not know	15 (53.6%)	13 (46.4%)		
Medical costs How do you think AI in ophthalmology will affect medical costs?			1.31	0.519
Reduce costs	76 (61.8%)	47 (38.2%)		
Increase costs	16 (51.6%)	15 (48.4%)		
I do not know	25 (55.6%)	20 (44.4%)		
Necessity of legislation Do you think the use of AI in ophthalmology should be regulated by legislation?			4.76	0.092
Yes	90 (75.3%)	67 (42.7%)		
No	18 (78.3%)	5 (21.7%)		
I do not know	9 (47.4%)	10 (52.6%)		
General impression Do you have a generally favorable impression of using AI in ophthalmology?			10.78	0.005
Yes	105 (63.4%)	60 (36.4%)		
No	7 (46.7%)	8 (53.3%)		
I do not know	5 (26.3%)	14 (73.7%)		
Interest in topic Are you interested in the topic of AI in ophthalmology?			3.88	0.143
Very interested	88 (63.3%)	51 (36.7%)		
Somewhat interested	27 (48.2%)	29 (51.8%)		
Not interested	2 (50.0%)	2 (50.0%)		
Accuracy Do you think AI in medicine will deliver accurate diagnoses?			6.59	0.037
Yes	75 (66.4%)	38 (33.6%)		
No	18 (52.9%)	16 (47.1%)		
I do not know	24 (46.2%)	28 (53.8%)		

Table 3 presents the responses of participants on their opinions of which tasks they think the AI chatbot could be most useful in their practice. There was no difference between ChatGPT users and non-users in their responses.

**Table 3:** Participants' opinions in which tasks do they think that the AI chatbot could be most useful in their practice. AI: artificial intelligence; N: number

Task	N(%)
Identifying early signs of eye diseases	109 (22.2%)
Providing decision support in treatment planning	92 (18.7%)
Monitoring patients' progress	100 (22.3%)
Answering patient questions	89 (18.1%)
Scheduling appointments	102 (20.7%)

Table 4 presents the responses to concerns about using an AI chatbot in clinical practice. There was no difference between ChatGPT users and non-users in their responses.

**Table 4:** concerns raised by participants about the use of AI chatbots in clinical practice AI: artificial intelligence; N: number

Concerns	N(%)
Liability issues	78 (19.0%)
Privacy concerns	85 (20.7%)
Informed consent and Ethical issues	54 (13.2%)
Accuracy of diagnosis	111 (27.1%)
Trustworthiness of the chatbot and information bias	82 (20.0%)

## Discussion

Over the recent past (months), there has been an increased interest in AI in medicine. Ophthalmologists, regardless of their gender, age, years of experience, and nationality, are keen on the development and use of AI in ophthalmology. In fact, the prevalence of ChatGPT use was 59% in our study. This showcases a significant acceptance for introducing new technologies in ophthalmology.

In this study, most participants from both cohorts - ChatGPT users and non-users - expressed a belief in the potential benefits of the application of AI in ophthalmology. Specifically, 62% of ChatGPT users and 38% of non-users viewed AI as useful, 63% of users and 37% of non-users considered it efficient, 66% of users and 34% of non-users regarded it as accurate, and 63% of users and 37% of non-users believed it could enhance the quality of medical services provided. Notably, comparison of physicians with less than five years of experience to those with over 15 years of experience indicated no difference in the proportion of those using ChatGPT (60% and 56%, respectively).

Concerns about AI supplanting physicians and surgeons appear unfounded, given the current limitations of AI models. These models lack the capacity to learn from their environment like humans do and struggle to adapt when situations deviate from their programmed algorithms [11]. Consequently, our analysis suggests that AI's efficacy and reliability in ophthalmology can be maximized when used in conjunction with human physicians rather than as a replacement.

Additionally, 63% of ChatGPT users and 37% of non-users believe that the patients are optimistic about the potential use of AI in ophthalmology. Although the use of AI seems encouraging and may aid physicians in achieving a faster and perhaps more accurate diagnosis to some extent, we believe that patients will still prefer to be diagnosed, and managed by a physician rather than a machine, which also supports the fact that it is probably impossible for machines to replace physicians and surgeons.

Incorporating AI into ophthalmology presents an initial financial challenge for healthcare institutions [12]. Nevertheless, this investment has the potential to yield long-term cost reductions [13,14]. AI, in collaboration with clinicians, could expedite and enhance the accuracy of diagnoses, potentially decreasing the frequency of patient visits and associated costs [11,14,15]. In our research, a significant portion of participants - 62% of ChatGPT users and 38% of non-users - anticipate that implementing AI in ophthalmology could decrease overall healthcare expenditures. Despite the initial financial outlay, this perspective underscores the potential economic benefits of integrating AI into medical practices.

Seventy-five percent of ChatGPT users and 43% of ChatGPT non-users believe that AI models should be regulated. The higher the accuracy of the information being fed into AI models, the more reliable the outcomes are. Therefore, Al models should be trained and supervised by trustworthy authorities in the field, ensuring no conflict of interest and information bias.

Despite the advances of ChatGPT in research, a previous study highlights the mixed acceptance of ChatGPT among physicians. While some appreciate its ability to improve efficiency, others have concerns about data privacy, potential biases in generated content, and the need for human oversight. To address these concerns, it is essential to establish guidelines and best practices for using ChatGPT in medical articles and to ensure that the generated content is reviewed by medical experts [16]. In our study, 20% had privacy concerns with the use of AI Chatbots.

Furthermore, physicians should know how AI models work to understand their limitations. AI does not take away from the important role of physicians, and complete dependence on AI models is not applicable. It is also essential for authors to disclose the use of AI tools when preparing manuscripts to maintain transparency and scientific integrity [17].

Limitations

The low number of participants and the unequal distribution in age and gender may have affected our results. Besides, developing a standardised questionnaire in the future would lead to more accurate assessment of the acceptance of ChatGPT use.

## Conclusions

The study reveals a growing acceptance of AI, especially ChatGPT, among ophthalmologists, with 59% of participants using it in clinical practice. AI is appreciated for its utility in enhancing medical services, decision support, and patient education, though concerns about privacy, potential job displacement, and the need for human oversight were noted. Most respondents believe AI can improve efficiency and accuracy in healthcare but emphasize its role as a supportive tool rather than replacing human expertise.

Regulatory guidelines and ethical considerations are crucial for the responsible use of AI in healthcare. There is a general consensus on the need for a balance between AI capabilities and human judgment, with an optimistic view that AI integration, while initially costly, could lead to long-term reductions in healthcare expenses. This study underscores the potential of AI to revolutionize ophthalmology, provided it is implemented cautiously and transparently.

## Appendices

Acceptance of the Use of Artificial Intelligence chatbot (ChatGPT) in ophthalmology Among physicians: A Questionnaire Survey

Dear Participant,

You are invited to participate in our study on the acceptance of an AI chatbot (ChatGPT) in ophthalmology among physicians. Your voluntary participation will help us understand your attitudes towards this technology. Your responses will be kept confidential and anonymous.

The survey will gather information on usefulness, efficiency, and impact on medical services, as well as tasks in which the AI chatbot could be most useful for physicians and potential impact on medical costs.

Thank you for your participation.

**Table 5:** Questions asked to participants.

Demographics:	
1-What is your age?	
	Under 30
	30-39
	40-49
	50-59
	Over 60
2- What is your Gender?	
	Male
	Female
3- What is your Nationality?	
	Saudi
	Non-Saudi
4- How many years of experience do you have in ophthalmology?	
	Less than 5 years
	5-10 years
	10-15 years
	More than 15 years
AI (ChatGPT) experience questionnaire:	
1-Have you ever used (ChatGPT) AI?	
	Yes
	No
2- Usefulness: Do you think that AI in Ophthalmology will be useful?	
	Yes
	No
	I don't know
3- Efficiency: If AI is used in Ophthalmology, do you think doctors could provide services more efficiently?	
	Yes
	No
	I don't know
4- Better medical services: Would using AI in Ophthalmology lead to better medical services?	
	Yes
	No
	I don't know
5- Mastery: Could ophthalmologist quickly master the use of AI in medicine?	
	Yes
	No
	I don't know
6- User-friendliness: Could ophthalmologist easily operate AI in medical settings?	
	Yes
	No
	I don't know
7- Expectations of others: Do you think people around you are optimistic about the potential of AI in ophthalmology?	
	Yes
	No
	I don't know
8- Expectations among patients: Do you think patients are optimistic about the potential of AI in ophthalmology?	
	Yes
	No
	I don't know
9- Brand impact: Are your views on AI in ophthalmology shaped by the businesses (or brands of such businesses) that developed the AI for ophthalmology?	
	Yes
	No
	I don't know
10- Knowledge of AI in other contexts: Do you know much about the use of AI in contexts other than ophthalmology?	
	Yes
	No
	I don't know
11- Knowledge of AI in medicine: Do you know much about the use of AI in ophthalmology?	
	Yes
	No
	I don't know
12- Medical costs: How do you think AI in ophthalmology will affect medical costs?	
	Reduce costs
	Increase costs
	I don't know
13- Necessity of legislation: Do you think the use of AI in ophthalmology should be regulated by legislation?	
	Yes
	No
	I don't know
14- General impression: Do you have a generally favorable impression of the use of AI in ophthalmology?	
	Yes
	No
	I don't know
15- Interest in topic: Are you interested in the topic of AI in ophthalmology?	
	Very interested
	Somewhat interested
	Not interested
16- Accuracy: Do you think AI in medicine will deliver accurate diagnoses?	
	Yes
	No
	I don't know
17- In which tasks do you think the AI chatbot could be most useful for you?	(multiple choices)
	Identifying early signs of eye diseases
	Providing decision support in treatment planning
	Monitoring patients' progress
	Answering patient questions
	Scheduling appointments
	Other:
18- What concerns do you have about using an AI chatbot in your clinical practice?	
	Liability issues
	Privacy concerns
	Informed consent
	Accuracy of diagnoses
	Trustworthiness of the chatbot
	Other:
